# Closing-opening wedge osteotomy for thoracolumbar traumatic kyphosis

**DOI:** 10.1186/s40001-014-0059-3

**Published:** 2014-11-01

**Authors:** Xiang Li, Junwei Zhang, Hehu Tang, Zhen Lu, Shizheng Chen, Yi Hong

**Affiliations:** School of Rehabilitation Medicine, Capital Medical University, Beijing, 100068 China; Department of Spine Surgery, Beijing Bo’ai Hospital, China Rehabilitation Research Center, Beijing, 100068 China

**Keywords:** Closing-opening wedge osteotomy, Posttraumatic kyphosis, Spinal osteotomy, Thoracolumbar spine

## Abstract

**Background:**

Surgical treatment modalities for post-traumatic kyphosis (PTK) remain controversial. Like vertebral column resection, closing-opening wedge osteotomy (COWO) can achieve satisfactory results for kyphosis with multiple etiologies. However, few studies have assessed this procedure for PTK. Our purpose was to evaluate the radiographic and clinical outcomes of COWO in a selected series of patients with PTK via a single posterior approach.

**Methods:**

In this retrospective case series, seven patients with symptomatic PTK in the thoracolumbar spine were reviewed. Five patients underwent surgery at the time of initial injury, and the other two initially underwent conservative treatment. All seven patients underwent COWO procedures through a single posterior approach. The Cobb angle was assessed preoperatively, postoperatively, and at the final follow-up. A visual analog scale (VAS) and the American Spinal Injury Association scale were used to evaluate back pain and neurological function preoperatively and at final follow-up, respectively. Operation-associated complications were also recorded.

**Results:**

The mean follow-up period was 34.3 months (range, 24 to 43 months). The mean kyphotic angle was significantly (*P* <0.05) reduced from 57.7° (range, 36° to 100°) preoperatively to 8° postoperatively (range, −12° to 50°). The mean VAS improved from 5.9 to 2.1 (*P <*0.05). Three patients exhibited improved neurological function. Bony fusion was achieved in all patients. No significant correction loss or permanent complication was noted.

**Conclusions:**

Though technically demanding, COWO via a single posterior approach can provide satisfactory outcomes for selected patients with PTK. Additional studies are required to improve patient selection and outcomes for this condition.

## Background

The thoracolumbar spine is a common site of spinal fractures [1]. Patients with missed fractures or initial treatment failure may be at risk of developing late post-traumatic kyphosis (PTK) [2]. Surgical treatment is indicated for PTK patients with progressive deformity, refractory back pain, or deterioration of neurological status, and such operations are challenging for spine surgeons [1-7].

Unlike Scheuermann’s kyphosis or ankylosing spondylitis, patients with PTK typically have short, angular curves with few involved segments and can always maintain overall sagittal balance [3,8]. Three-column posterior osteotomies, including pedicle subtraction osteotomy (PSO) and vertebral column resection (VCR), are more effective for correcting PTK than a posterior column-only osteotomy, such as Smith-Petersen Osteotomy [8,9].

Closing-opening wedge osteotomy (COWO) is a surgical technique similar to VCR [10,11]. The vertebral body and adjacent discs above and below are simultaneously excised through a single-stage posterior approach. Anterior reconstruction is achieved by the insertion of a metal mesh or a strut autograft, which can both improve the kyphosis correction and reduce the incidence of dural buckling [11]. Satisfactory results have been achieved by using COWO to treat kyphosis with multiple etiologies [6,11,12]. In PTK cases, it has been reported that transpedicular osteotomy was effective [13]. However, more studies focusing on the use of COWO to treat PTK are required [6].

The purpose of this study was to evaluate the radiographic and clinical outcomes in seven patients who underwent COWO for PTK correction in our institution. We also discuss the unique characteristics of PTK.

## Methods

The study conformed to World Medical Association Declaration of Helsinki (June 1964) and subsequent amendments. No ethical approval was needed for the present study. From April 2006 to October 2010, seven consecutive patients with symptomatic thoracolumbar PTK were surgically treated in our department. There were 3 men and 4 women with an average age of 36.3 years (range, 20 to 57 years). The mean delay between the initial injury and correction surgery was 87.9 months (range, 6 months to 54 years). According to the AO fracture classification, the initial fracture patterns system included A3.1 in one patient, A3.3 in two patients, B1.2 in two patients, and C1.2 in one patient [14]. The fracture pattern for one patient (case 4) was not clear because the injury had occurred 54 years earlier. Conservative treatment was initially performed in two patients. The other five patients had undergone surgery at the time of injury. The initial treatments included simple laminectomy without instrumentation in two patients, posterior laminectomy and stabilization with interspinous wires in one patient, and posterior reduction and long-segment pedicle screw instrumentation in two patients. The last two patients developed deep wound infections and underwent implant removal 1 and 4 months later, respectively. None of the seven patients were initially treated at our institution. All the patients presented with neurological deficits; grade A in 1 patient, grade B in 1 patient, grade C in 3 patients, and grade D in 2 patients, according to the American Spinal Injury Association (ASIA) scale [15]. All the patients complained of moderate to severe back pain according to a visual analog scale (VAS). The patient characteristics and medical histories are summarized in Table 1.

**Table 1:** **Preoperative demographic and injury data**

**Case**	**Sex**	**Age**	**Injury level**	**Injury pattern**	**Initial treatment**	**Time from injury (months)**
1	F	42	L1	B1.2	Posterior laminectomy without instrumentation	92
2	M	43	T12	A3.3	Posterior laminectomy and stabilization with interspinous wires	43
3	F	30	T11	B1.2	Posterior laminectomy without instrumentation	6
4	M	57	L2	Unclear	Conservative treatment	432
5	F	26	T12	A3.3	Conservative treatment	19
6	F	36	T12	A3.1	Posterior fixation	7
					Deep wound infection after surgery and implant removal	
7	M	20	L1/L2	C1.2	Posterior fixation	16
					Deep wound infection after surgery and implant removal	

### Surgical technique

All the patients underwent surgery under general anesthesia. Spinal cord monitoring or wake-up testing was only performed for selected patients with ASIA grade C or D. All surgeries were performed by senior author YH.

The surgeries were performed according to the method described by Kawahara et al. [11], with some modifications. Briefly, a patient under general anesthesia was placed in a prone position, and a midline incision was made to expose the osteotomy site and adjacent two to three segments above and below. Pedicle screws (CD HORIZON, Medtronic, Minneapolis, MN, USA, in six cases and TSRH, Danek, Memphis, TN in one patient) were inserted into the corresponding levels.

### Osteotomy procedure

In the upper lumbar region, the posterior elements, including the spinous process, bilateral lamina, transverse process, and the adjacent facet joints are excised to identify the involved pedicles. In the lower thoracic region, it is necessary to excise ribs 3 to 4 cm lateral to the costotransverse joint, the rib heads and transverse process at the corresponding level. After the above procedures, the affected pedicles are completely surrounded. Nerve roots beneath the involved pedicles are carefully dissected 3 to 4 cm lateral to the corresponding foramen. The segment nerve roots should be gently retracted to make space for obtaining access to the anterior column and osteotomy. In our experience, the operative area at the thoracolumbar region is wide enough to perform vertebrectomy from a posterior approach and it is not necessary to sacrifice the nerve root. The lateral aspect of the target vertebral body is then bluntly dissected from both sides through the plane between the anterior soft tissue and vertebral body. In the lower thoracic region, this procedure should be performed extrapleurally. Usually, the great vessels are surrounded by a thick layer of fibrous tissue and can be safely separated from the anterior aspect of the vertebral body. One unilateral temporary rod is fixed to maintain the spinal stability during the osteotomy procedure. With pedicle guidance, the corresponding vertebral body and adjacent discs are excised from the lateral direction with an osteotome, rongeur, curette, and high-speed bur. The integrity of the posterior wall cortex and the end plates of the adjacent vertebral body should always be preserved. In our protocol, it is not mandatory to totally remove the vertebra as described by Tomita et al. [16]*.* However, the anterior part of the vertebral body must be carefully removed and cleaned so that the opening of the anterior column can be achieved during the correction maneuver. Following dissection of the adhesion between the ventral dura and posterior wall cortex with a Penfield dissector, the residual posterior wall cortex is pushed into the anterior intervertebral gap and then the circumferential decompression of the spinal cord is completed.

### Correction procedure

In cases 1 to 4, two-step closing-opening wedge osteotomy and correction was performed. At the beginning of the correction maneuver, a contoured rod (Rod B) which was slightly less kyphotic than the original deformity was attached to the screws on the opposite side, while loosening the connection of screws of the temporary rod (Rod A). Rod A was then removed and bended to a slightly less kyphotic degree than Rod B and re-attached to the screws, while carefully removing Rod B. The above closing-wedge procedures were repeated by the surgeon and assistant alternately and stopped at the first evidence of dura buckling. The residual intervertebral gap was measured, and a Pyramesh titanium mesh (Medtronic, Memphis, USA) packed with autogenous bone grafts obtained from the osteotomy procedure was carefully inserted into the intervertebral gap*.* With shifting of the fulcrum to the interface between the titanium mesh and end plates above and below, the residual kyphosis was corrected by gradually compressing the posterior instrumentation and/or *in situ* contouring technique if necessary. This maneuver can both guarantee the opening of the anterior column and maintain the length of the spinal cord.

For cases 5 to 7 with relatively severe kyphosis, the intervertebral gap after vertebrectomy was fairly large. In order to prevent sagittal translation during the correction procedure, a titanium mesh (PYRAMESH, Medtronic) packed with autogenous bone was directly inserted into the intervertebral gap after vertebrectomy. The kyphotic deformity was corrected by the same method stated above with an interface hinge between the titanium mesh and the end plates above and below. This procedure can achieve anterior column opening and posterior column closing in one step.

The remaining bone grafts were placed around the cage to fill the gap. The drainage tube was placed beneath the muscle, and the wound was closed in layers.

### Postoperative management

Postoperatively, the patients were allowed out of bed with a custom-molded thoracolumbosacral orthosis 1 week after operation if no evidence of continuous cerebrospinal fluid leakage was noted. The patients were required to wear the thoracolumbosacral orthosis for 3 to 4 months.

### Clinical and radiological assessments

The medical records of the COWO procedure, including operative time, blood loss, and perioperative complications were recorded.

Radiographs were obtained preoperatively, postoperatively, and at the final follow-up. Follow-up X-ray films, including an A-P film, lateral film, and flexion/extension films were obtained. Kyphotic angles were measured by Cobb’s method, which was from the superior endplate of the vertebra above the injured level to the inferior endplate of the vertebra below the injured level. Positive and negative values indicated kyphosis and lordosis, respectively. The status of fusion was classified into solid union, probable union, and nonunion according to Suk’s criteria [17]*.* Back pain severity was evaluated by a visual analog scale (VAS) preoperatively and at the final follow-up. The ASIA scale was used to assess neurological function at the same time points.

### Statistical analysis

Data were statistically analyzed using SPSS 11.5 software (SPSS Inc., Chicago, IL, USA). Wilcoxon signed rank tests were used to assess kyphotic angle differences before and after surgery or at the final follow-up. For VAS, results were evaluated by comparing the patient’s preoperative score to that at the final follow-up with the same method. *P* <0.05 was considered statistically significant.

## Results

All the patients tolerated the operations well and had a mean follow-up period of 34.3 months (range, 24 to 43 months). The mean blood loss was 3,071 mL (range, 2,200 to 4,100 mL), and the mean operative time was 376 minutes (range, 320 to 430 minutes). The kyphotic angle improved from a preoperative mean of 57.7° (range, 36° to 100°) to a postoperative mean of 8° (range, −12° to 50°), which was statistically significant (*P* <0.05). The follow-up kyphotic angle was 11.6°, which corresponded to a mean 3.6° correction loss, but the angle was still significantly improved compared to the mean preoperative value (*P* <0.05).

One patient (case 4, ASIA grade D) experienced a transient postoperative deterioration of neurological function, but her symptoms improved to preoperative status after 1 month of conservative treatment. No permanent neurological function deterioration was noted. Intraoperative dural tears occurred in two patients (cases 1 and 6), and postoperative cerebrospinal fluid leakage occurred in both cases. These symptoms resolved spontaneously, and no deep wound infection was identified. No other perioperative complications occurred.

All the patients reported significant back pain relief at the final follow-up. The mean VAS score improved from 5.9 preoperatively (range, 4 to 7) to 2.1 at the final follow-up (range, 1 to 3), which was statistically significant (*P* <0.05). Postoperative neurological function improvement was noted in three patients. At the final follow-up, one patient (case 7) improved from ASIA grade B to C, and two patients (cases 1 and 3) improved from ASIA grade C to D. All patients achieved bony fusion, and no implant failure was noted.

The clinical and radiological results are summarized in Table 2.

**Table 2:** **Treatment, complications, and follow-up data**

**Cases**	**Fusion levels**	**Blood loss (ml)**	**Operative time (min)**	**Follow-up time (month)**	**Kyphotic angle (degree)**				**ASIA grade**		**Follow-up VAS**		**Complications**
					**Pre-op**	**Post-op**	**Follow- up**	**Loss of correction**	**Pre-op**	**Follow-up**	**Pre-op**	**Follow-up**	
1	T11-L3	3500	430	36	54	−2	0	2	C	D	6	1	Dural tear
2	T10-L2	3300	400	41	48	2	7	5	C	C	4	2	
3	T9-L1	2600	345	43	36	10	12	2	C	D	5	2	
4	T10-L5	3000	425	28	100	50	56	6	D	D	7	3	Transient neurological deficit
5	T11-L3	2800	385	38	52	−12	−8	4	D	D	5	2	
6	T10-L2	2200	330	30	62	−4	−2	2	A	A	7	3	Dural tear
7	T9-L4	4100	320	24	52	12	16	4	B	C	7	2	

ASIA, American Spinal Injury Association; VAS, Visual Analog Scale.

### Illustrative cases presentation

#### Case 3

**Figure 1:**
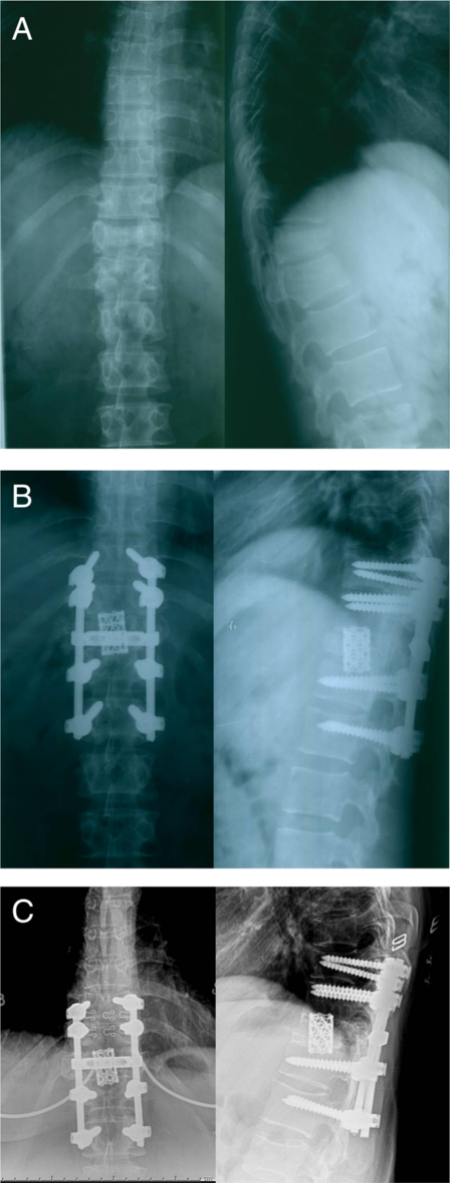
**Radiographs of case 3. (A)** Preoperative X-ray films. Left: Preoperative A-P film showed absence of posterior element at T11 level which indicated that laminectomy had been performed. Right: The lateral film showed anterior collapse of T11 vertebral body and local kyphosis of 36°. **(B)** Immediately postoperative X-ray films: A closing-opening wedge osteotomy at T11 level and T9-L1 pedicle screw instrumentation (CD HORIZON, Medtronic, Minneapolis, MN, USA) and fusion were performed. The kyphotic angle was corrected to 10°. **(C)** Follow-up X-ray films: Bony fusion was achieved and there was only 2° of correction loss at 3 years follow-up.

This 30-year-old female patient sustained a T11 burst fracture (AO classification B1.2) in a motor vehicle accident with neurological deficit. She underwent posterior laminectomy without instrumentation as the initial treatment at another hospital. Kyphosis and back pain developed gradually after the surgery. When she came to our outpatient department 6 months after the initial injury, she complained of severe back pain and presented with T10 spinal cord injury (ASIA grade C). She could not stand and walk for any prolonged period because of back pain. The preoperative kyphotic angle was 36°. To correct the deformity, a closing-opening wedge osteotomy at T11 level and T9-L1 pedicle screw instrumentation (CD HORIZON, Medtronic, Minneapolis, MN, USA) and fusion were performed. After surgery the local kyphosis improved to 4° and significant pain relief was noted. No significant loss of correction or implant failure was reported at 3 years follow-up. The neurological function improved from ASIA grade C preoperatively to grade D at final follow-up (Figure 1).

#### Case 4

**Figure 2:**
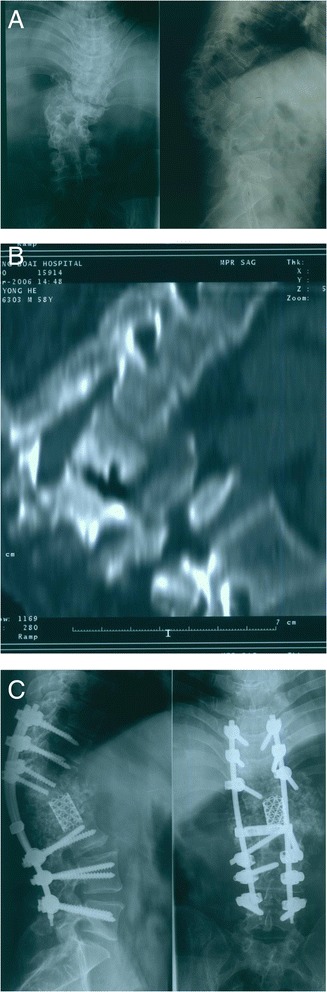
**Radiographs of case 4. (A)** Preoperative X-ray films. Left: A-P film showed scoliosis of 20°. Right: Lateral film showed significant collapse of L1 vertebral body and kyphosis of 100°. **(B)** Sagittal computed tomography scan preoperative. **(C)** Postoperative X-ray films. Vertebrectomy of L1 and L2 from posterior approach and one-step closing-opening wedge osteotomy with pedicle screws instrumentation (CDH + TSRH, Medtronic, Memphis, USA) from T9-L4 were performed. Left: Lateral film. Kyphosis was corrected to 50°. Right: A-P film. Scoliosis was corrected to 10°.

This 57-year-old male patient, according to his own personal statement, sustained a falling injury when he was 3 years old but did not receive any treatment due to economic problems. The kyphotic deformity of lumbar spine developed progressively. When he came to our outpatient clinic, he presented with severe cosmetic problems in the lumbar spine and weakness of bilateral iliopsoas and quadriceps. The preoperative pain was graded as 7, which influenced his normal sleep. The preoperative X-ray showed significant collapse of L2 segment and local kyphotic angle was 100°. The patient underwent vertebrectomy of L1 and L2 from posterior approach and one-step closing-opening wedge osteotomy with pedicle screws instrumentation (CDH + TSRH, Medtronic, Memphis, USA) from T9-L4. After surgery, the kyphotic angle improved to 50° and pain relieved significantly. The patient experienced transient weakness of iliopsoas after surgery and recovered to preoperative status after 1 month of conservative treatment (Figure 2).

#### Case 7

**Figure 3:**
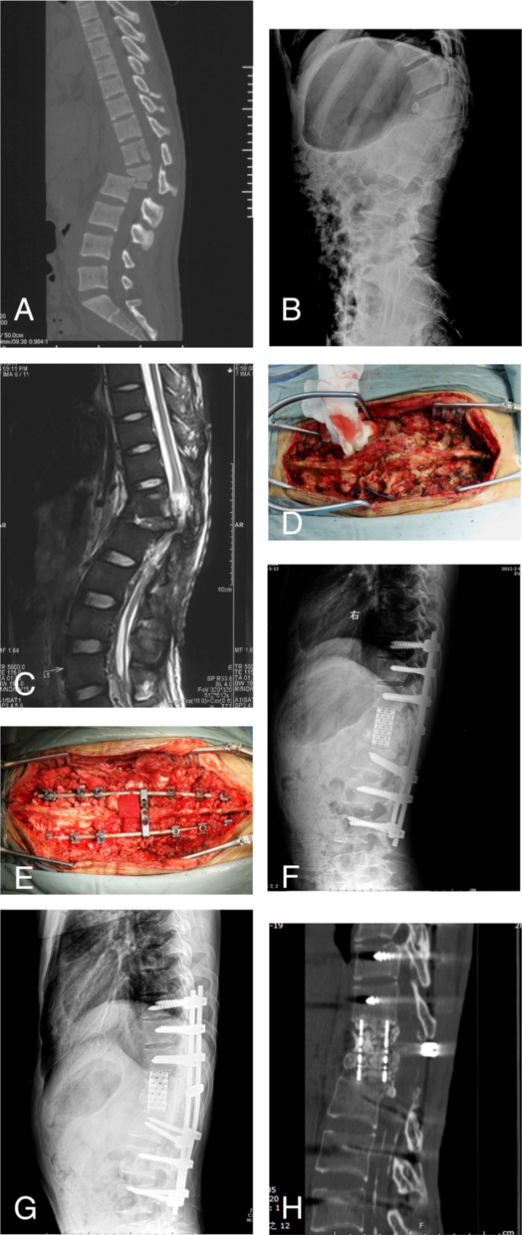
**Radiographs and photographs of case 7. (A)** Sagittal computed tomography scan of the initial injury showed dislocation at L1/L2 levels and significant comminution of the L1 vertebral body. **(B)** Preoperative lateral X-ray film: Collapse of the anterior column after implant removal and local kyphosis of 52°. **(C)** Preoperative MRI T2-weighted film. Intraoperative photographs of thoracolumbar spine before **(D)** and after **(E)** correction. **(F)** Immediately postoperative lateral radiograph: The kyphosis was corrected to 12°. **(G)** Lateral film at 2-year follow-up: Good maintenance of the correction and 4° of correction loss was noted. **(H)** Computed tomography scan at 2-year follow-up: Solid bony intervertebral fusion was achieved.

This 20-year-old male patient suffered a fracture and dislocation at L1/2 segments in a falling injury. The neurological status was T10 spinal cord injury (ASIA grade C) at initial injury. He was treated with posterior long-segment pedicle instrumentation immediately after injury in another hospital. Unfortunately, uncontrolled deep wound infection occurred one month after the surgery, which made it necessary to remove all the implant and perform debridement and irrigation. Finally, the wound healed, but the collapse and kyphosis at the injury level developed progressively. When the patient was admitted to our ward at 16 months after the initial injury, he presented with deterioration of the neurological function (ASIA grade B) and severe back pain, which influenced normal sitting position and rehabilitation. A closing-opening wedge osteotomy at T12/L1 levels and T9-L4 pedicle screw fixation (CD HORIZON, Medtronic, Minneapolis, MN, USA) and fusion were performed to correct the kyphotic deformity. The kyphosis improved from 52° preoperatively to 12° postoperatively and had 4° of loss of correction at 2-year follow-up. The neurological function improved from ASIA grade B preoperatively to ASIA grade C and significant pain relief was also noted at final follow-up (Figure 3).

## Discussion

The correction of kyphosis can be achieved by an anterior approach, posterior approach, or a combined anterior-posterior approach. Compared to the posterior approach, the anterior approach has advantages including direct decompression of the spinal canal, better correction maintenance, and more biomechanical stability. However, disadvantages associated with the anterior approach, such as it being a complicated surgical approach, massive trauma, and high risk of perioperative complication, limit the extensive application of this wide-open procedure [18]*.* EI-Sharkawi et al. [4] compared the surgical outcomes of PSO with anterior corpectomy and plating in patients with PTK. Significantly better results were observed in the PSO group in terms of correction angle (29.8° for PSO group and 22° for the anterior corpectomy and plating group) and improvement in VAS score. A study by Suk et al. [1] also supported the use of PSO for the treatment of PTK compared with a combined anterior-posterior approach. Nowadays, the posterior-only approach has become the most commonly used method for the treatment of kyphosis.

Unlike kyphosis due to other etiologies, PTK is usually a regional kyphosis and is more likely to be a short, angular curve. This condition is more amendable to three-column osteotomies, including PSO and VCR, than a posterior column-only osteotomy such as PSO [3,8].

Recently, satisfactory mid- to long-term results have been achieved by employing PSO to treat sagittal imbalance due to multiple etiologies [1,4,19-21]. Kim et al. [21] reported the 5- to 8-year follow-up results of PSO for the treatment of sagittal balance in 35 patients and obtained a mean correction angle of 36.2° and significant improvement on the Oswestry disability index. No pseudarthrosis or permanent neurological deficits were noted. In a series of 27 patients who underwent PSO for the treatment of sagittal imbalance due to multiple etiologies, Bridwell et al. [19] reported an average 34.1° of increase of lordosis, 13.5 cm of improvement of the sagittal line, and significant improvement in Oswestry score with at least 2 years follow-up. A study by Brox et al. [20] had similar results.

However, PSO is not a complication-free procedure. Recently, Bridwell et al. [8] reviewed the literature and found that the incidence of neurological deficits with PSO was about 8%, though most deficits were transient. This complication is partially attributed to dural buckling when the kyphosis to be corrected is >40° [22]. Further, Bridwell et al. [23] analyzed complications associated with PSO in 33 patients with at least 2-year follow-ups; pseudarthrosis was noted in eight patients, including one case in the osteotomy site, which affected the clinical results. The authors strongly recommended anterior reconstruction for osteotomies performed through segments with a history of previous laminectomy, pseudarthrosis, or nonfusion. In a series of 67 patients, Ikenaga et al. [24] reported that 18 patients (26%) experienced kyphosis progression after the PSO procedure, and one-third of these 18 patients required revision surgery because of pain and neurological deterioration. Patients with compression fractures were more likely to develop adjacent segment collapse compared with patients with degenerative kyphosis. Auerbach et al. [25] reported that the overall rate of major medical and surgical complications in the PSO group (38%) was higher than that in the VCR group (22%), but this difference was not significant.

In the present study, five of the seven patients had thoracolumbar kyphosis >45°, and three of seven patients initially underwent laminectomy without rigid stabilization. Two of seven patients experienced uncontrolled deep wound infection and underwent implant removal within a few months of surgery. Pseudarthrosis occurred at the initial fracture site, and kyphosis developed progressively. Given the clinical characteristics mentioned above, we did not think PSO was appropriate for this group of patients.

COWO was first described by Kawahara et al. [11] and is useful for correcting severe kyphosis (>50°). This procedure involves resection of the vertebral body and adjacent discs above and below, all through a single-stage posterior approach. Greater kyphosis correction can be achieved without kinking the spinal cord by inserting a cage into the intervertebral gap to serve as a secondary fulcrum. Anterior support provided by the metal cage may also avoid the inherent complications associated with PSO procedure.

In the present study, we obtained an average 50.6° kyphosis correction without permanent neurological deficits in seven patients. Three patients achieved one ASIA grade improvement, and no significant loss of correction was noted at the final follow-up. These results are comparable to those described by others. Kawahara et al. [11] reported 49° kyphosis correction and 100% fusion rate in seven patients who underwent COWO for severe kyphosis with multiple etiologies. In a series of 19 patients who underwent this technique to correct moderate-to-severe PTK, Zeng et al. [6] achieved an 83.3% correction rate. VAS and Oswestry disability index improved from 10.0 and 27.4 preoperatively, to 2.3 and 12.2 at the final follow-up, respectively. In addition, the authors emphasized that patients with neurological deficits could also benefit from this procedure, regardless of the duration of the delay between the initial injury and the correction surgery. Rajasekaran et al. [12,26] also obtained satisfactory results by using this procedure to treat severe post-tubercular kyphotic deformities.

The decision to perform PSO or COWO/VCR is mainly dependent on the magnitude of kyphosis. Zeng et al. [6] suggested that COWO should be applied if the kyphotic angle is >45°. Zhang et al. [7] performed VCR and achieved satisfactory results when the effective regional deformity was >60°.

Based on our experience, we think that factors besides the kyphotic angle should be considered when choosing an appropriate treatment for symptomatic PTK. Recently, Schoenfeld et al. [2] reviewed the literature and reported that severe burst fractures and flexion-distraction were the most common PTK-associated injury patterns, which we also observed in the present study. As the largest avascular structures in the adult human body, intervertebral discs have limited ability to heal after injury. Studies by Haschtmann et al. [27] and Heyde et al. [28] showed that endplate fracture could induce apoptosis in intervertebral disc cells, which may accelerate the degenerative processes. Oner et al. [29] reported that the primary correction loss after surgical treatment for thoracolumbar burst fractures occurred at the intervertebral disc space rather than in the vertebral body. Jean et al. [9] also found that adjacent disc damage prevented full kyphotic deformity correction in the lower lumbar spine. More importantly, disc damage may be related to pain in patients with PTK [30]. Therefore, it is our preference to perform COWO, which involves resection of the injured vertebral body and adjacent discs to correct symptomatic PTK, especially when the initial injury patterns are severe burst fracture and flexion-distraction. That is why we performed this procedure for case 3, who had a thoracolumbar kyphosis of only 36°. In addition, the surgical experience of the physician should be considered when choosing an appropriate treatment.

As a retrospective case series, the present report has some inherent limitations. The choice of treatment was mostly dependent on the surgeon’s personal experience, and it is likely that PSO is more suitable for certain patients with PTK. Treating PTK is challenging, and even an experienced physician can only manage a handful of patients with this condition. We were unable to quantify the factors influencing the surgeon’s decision to perform PSO or COWO/VCR because of the small sample size. Multicenter prospective studies comparing outcomes among different surgical treatments should be performed to improve patient selection and surgical outcomes.

## Conclusions

Unlike Scheuermann’s kyphosis or ankylosing spondylitis, patients with PTK usually have a short, angular curve, which is more likely to respond to three-column posterior osteotomy. Though technically demanding, the COWO procedure via a single posterior approach can achieve satisfactory surgical outcomes for selective patients with PTK. Larger studies are required to improve patient selection and surgical outcomes for this condition.

### Consent

Written informed consent was obtained from the patient for the publication of this report and any accompanying images.

## Abbreviations

ASIA: American Spinal Injury Association
COWO: Closing-opening wedge osteotomy
PSO: Pedicle subtraction osteotomy
PTK: Post-traumatic kyphosis
VAS: Visual analog scale
VCR: Vertebral column resection
